# Identification of Long Noncoding RNAs Involved in Eyelid Pigmentation of Hereford Cattle

**DOI:** 10.3389/fgene.2022.864567

**Published:** 2022-05-04

**Authors:** Eugenio Jara, Francisco Peñagaricano, Eileen Armstrong, Claudia Menezes, Lucía Tardiz, Gastón Rodons, Andrés Iriarte

**Affiliations:** ^1^ Unidad de Genética y Mejora Animal, Departamento de Producción Animal, Facultad de Veterinaria, Universidad de La República, Montevideo, Uruguay; ^2^ Department of Animal and Dairy Sciences, University of Wisconsin-Madison, Madison, WI, United States; ^3^ Laboratorio de Endocrinología y Metabolismo Animal, Facultad de Veterinaria, Universidad de La República, Montevideo, Uruguay; ^4^ Laboratorio de Biología Computacional, Departamento de Desarrollo Biotecnológico, Instituto de Higiene, Facultad de Medicina, Universidad de La República, Montevideo, Uruguay

**Keywords:** eye cancer, lncRNAs, noncoding genetic elements, strand-specific RNAseq, beef cattle

## Abstract

Several ocular pathologies in cattle, such as ocular squamous cell carcinoma and infectious keratoconjunctivitis, have been associated with low pigmentation of the eyelids. The main objective of this study was to analyze the transcriptome of eyelid skin in Hereford cattle using strand-specific RNA sequencing technology to characterize and identify long noncoding RNAs (lncRNAs). We compared the expression of lncRNAs between pigmented and unpigmented eyelids and analyzed the interaction of lncRNAs and putative target genes to reveal the genetic basis underlying eyelid pigmentation in cattle. We predicted 4,937 putative lncRNAs mapped to the bovine reference genome, enriching the catalog of lncRNAs in *Bos taurus*. We found 27 differentially expressed lncRNAs between pigmented and unpigmented eyelids, suggesting their involvement in eyelid pigmentation. In addition, we revealed potential links between some significant differentially expressed lncRNAs and target mRNAs involved in the immune response and pigmentation. Overall, this study expands the catalog of lncRNAs in cattle and contributes to a better understanding of the biology of eyelid pigmentation.

## 1 Introduction

Long noncoding RNAs (lncRNAs) are transcripts longer than 200 bp that do not code for functional proteins (Qureshi et al., 2010). They constitute a highly heterogeneous class of RNAs, including enhancer RNAs, antisense transcripts, and intergenic lncRNAs (Boon et al., 2016). They are transcribed and processed like messenger RNAs (mRNAs): lncRNAs are 3′ polyadenylated, 5′ capped, and multiexonic (Carninci, 2005; Derrien et al., 2012; Li et al., 2019). They have been studied and characterized in various animal species, including mice (Kadakkuzha et al., 2015), humans (Gibb et al., 2011), sheep (Bakhtiarizadeh et al., 2016), goats (Ren et al., 2016; Ling et al., 2017), chickens (You et al., 2019) and cattle (Huang et al., 2012; Weikard et al., 2013; Billerey et al., 2014; Koufariotis et al., 2015; Kern et al., 2018). They are usually expressed at low levels and are under weaker selective constraints than protein-coding genes (Derrien et al., 2012). LncRNAs are poorly conserved among different species compared to mRNAs, and their expression is tissue-specific (Pang et al., 2006; Cabili et al., 2011; Derrien et al., 2012; Kern et al., 2018). Given these characteristics, lncRNAs are regarded as transcriptional “noise” (Chakalova et al., 2005; Ma et al., 2013). In fact, the role of lncRNAs is poorly understood. LncRNAs have been shown to be associated with various critical biological processes, mainly through the regulation of gene expression (Clemson et al., 1996; Gupta et al., 2010; Ma et al., 2013; Weikard et al., 2013; Dempsey and Cui, 2017). LncRNAs can act as signals, decoys, guides and scaffolds to regulate gene expression at the pretranscription, transcription, and post-transcription stages, regulating gene expression in *trans* and *cis* (Kornienko et al., 2013; Li et al., 2019). In genome-wide association studies (GWAS), the vast majority of significant single nucleotide polymorphisms (SNPs) are located in non-coding regions (Laurent et al., 2014; Goddard et al., 2016), suggesting that noncoding transcripts could play a more important role than expected, being directly involved in phenotypic variation (Kosinska-Selbi et al., 2020). Notably, the catalog of lncRNAs in livestock animals compared to humans and mice is far from complete (Kern et al., 2018; Kosinska-Selbi et al., 2020).

The study of coat and skin color in cattle has both economic and scientific interest. Several ocular pathologies, such as ocular squamous cell carcinoma, also known as eye cancer, and infectious keratoconjunctivitis, also known as pink eye, have been associated with low pigmentation of the eyelids (Heeney and Valli, 1985; Anderson, 1991). These ocular pathologies have a considerable economic impact since affected animals suffer weight loss and are underpaid at slaughter. Skin pigmentation is a complex, polygenic trait (Cichorek et al., 2013; Ainger et al., 2017). This trait is the result of differences in biochemical processes and the activity of melanocytes (Solano, 2014), which produce two types of melanin, eumelanin (black/brown) and pheomelanin (red/yellow) (Le Pape et al., 2008). The lack of eyelid pigmentation in Hereford cattle is the result of a genetic background that impact melanocyte development, including cell migration. This genetic background may be caused by variations in the expression of gene *KIT* during embryo development, resulting in impaired migration of melanocyte precursors to the region around the eyes (Grichnik, 2006). The receptor tyrosine kinase *KIT* and its ligand (*KITLG*) play an important role in the development of melanocytes, including migration, survival, proliferation, and differentiation (Grichnik, 2006; Mort et al., 2015; Ainger et al., 2017). Gene *KITLG* has been reported as a candidate gene affecting both eye area pigmentation and eyelid pigmentation in cattle (Pausch et al., 2012; Jara et al., 2022). There are many genes specifically expressed in melanocytes, such as *TYR*, *TYRP1*, *DCT*, *PMEL*, *MITF*, and *MLANA* (D’Mello et al., 2016; Ainger et al., 2017). The regulation of these important genes by lncRNAs has not been studied in relation to eyelid pigmentation in cattle. In recent years, the importance of lncRNAs in the biology of the skin (Wan and Wang, 2014) in different livestock species, including sheep (Yue et al., 2016), goats (Ren et al., 2016), and cattle (Weikard et al., 2013), has been demonstrated. In cattle, 4,848 potential lncRNAs were identified in a study that compared regions of pigmented and non-pigmented skin (body spots) (Weikard et al., 2013). The authors concluded that the transcription pattern of bovine skin is complex and suggested a possible functional relevance of new transcripts, including lncRNAs, in the modulation of pigmentation. The catalog of lncRNAs involved in skin pigmentation in cattle is not very extensive and limited to intergenic lncRNAs (Weikard et al., 2013).

The main objective of this study was to analyze the transcriptome of eyelid skin in Hereford cattle using strand-specific RNA sequencing (ssRNA-seq) to characterize and identify lncRNA possibly involved in eyelid pigmentation. Two contrasting groups were evaluated: steers with completely pigmented eyelids versus steers with no pigmentation in both eyelids (Jara et al., 2020). We evaluated the differential expression of lncRNAs between these two groups and analyzed the interactions between lncRNAs and putative target coding genes to reveal the genetic basis underlying this complex, economically relevant phenotype. Our study provides a valuable resource for the comprehension of lncRNAs, enriches the lncRNA catalog in cattle, and contributes to a better understanding of the molecular mechanisms underlying eyelid pigmentation.

## 2 Materials and Methods

### 2.1 Data

These RNA-seq data are available at the NCBI BioProject database with accession number PRJNA627111. The transcriptomes of 11 eyelid skin samples, five samples from 100% pigmented animals, and six samples from 0% pigmented animals were analyzed (Jara et al., 2020). The eyelid transcriptomes were generated using poly-A capture and strand-specific RNA sequencing in an Illumina HiSeq 2500 sequencing system. A total of 542,751,474 (38.5 G) clean reads were obtained. These reads were mapped to the latest cattle (*Bos taurus*) reference genome ARS-UCD1.2, using the software Hisat2 (v2.1.0) (Kim et al., 2015). The overall mapping rate ranged from 91 to 93%, and only uniquely mapped reads were considered (Supplementary Table S1). Transcriptomes were assembled for each sample using Cufflinks, and then, all the assemblies were merged into one using Cuffmerge.

### 2.2 Identification of Long NonCoding RNAs

Potential lncRNAs were identified using successive filters starting from all obtained transcripts. First, transcripts that presented the class code “ = “, “e”, “p”, and “c” from the output generated by Cuffcompare were filtered out (You et al., 2019). Transcripts with less than two exons with low expression levels (ten mapped reads per sample in at least five biological replicates were defined as the expression threshold) and shorter than 200 bp were also filtered out. DNA sequences of the transcripts were extracted using gffread (Trapnell et al., 2010). The sequences of known lncRNAs were downloaded from two multispecies lncRNA databases, ALDB (Domestic-Animal LncRNA Database) (Li A. et al., 2015) and NONCODE (Zhao Y. et al., 2016), which contain 8,250 and 23,515 cattle lncRNAs, respectively. BLASTn (version 2.2.31) (Altschul et al., 1990) was used to align the unannotated transcripts to lncRNAs in these two databases using stringent parameters (e-value ≤ 1 × 10^6^, coverage and identity ≥90%). Accurately aligned transcripts against either ALDB or NONCODE (5.0) were regarded as known lncRNAs. On the other hand, the transcripts that did not align with sequences in either ALDB or NONCODE were considered putative novel lncRNAs. This pipeline was adapted from Konsinska-Selbi et al. (2020).

### 2.3 Coding Potential Analysis

We calculated the coding potential of each transcript using three complementary tools: Coding Potential Calculator 2 (CPC2, version 0.1) (Kang et al., 2017), Coding-Potential Assessment Tool (CPAT, version 2.0.0) (Wang et al., 2013), and Predictor of long noncoding RNAs and messenger RNAs based on an improved k-mer scheme (PLEK, version 1.2) (Li et al., 2014). PLEK and CPC2 are based on the same support vector machine-based (SVM) classification model, while CPAT is based on a logistic regression (Wang et al., 2013; Antonov et al., 2019). All these programs are alignment-free tools and have been proven to be highly effective in discriminating lncRNAs (Han et al., 2016).

The CPAT program estimates coding probability scores. The optimum cutoff value for protein-coding probability is species-specific, and hence, the CPAT was trained using a set of 10,000 known bovine protein-encoding transcripts (*Bos*_taurus.ARS-UCD1.2.cds.all.fa, version 95) and a set of 10,000 bovine noncoding sequences (larger than 200 bases). The final reference dataset of noncoding RNAs comprised 37,695 sequences (5,930 from *Bos*_taurus.ARS-UCD1.2.ncrna.fa (version 95), 23,515 from NONCODEv5_cow.fa, and 8,250 from ALDB.cow.lincRNAs.v1.0.fa). Bovine protein-coding transcripts and noncoding sequences were extracted randomly from each annotation, following previously published studies (Billerey et al., 2014; Gupta et al., 2019). In brief, the two training sets were randomly split into ten different parts to perform a 10-fold cross-validation analysis. The cutoff value was selected to maximize specificity and sensitivity. On the other hand, PLEK uses a sliding window-based approach to analyze the transcripts based on a k-mer frequency distribution. PLEK was trained using the same set of sequences that were used for training the CPAT program.

Transcripts that displayed a CPC2 score lower than 0.5, a CPAT score lower than or equal to 0.36, and a PLEK score lower than 0 were considered noncoding genes and were used in subsequent analyses.

### 2.4 Gene Expression Analysis

Differentially expressed genes were identified using the R package DESeq2 (version 1.18.1) with default parameters (Love et al., 2014). Putative novel lncRNAs, known lncRNAs, and annotated protein-coding genes were all included in this statistical analysis. Only genes with at least ten reads per sample in at least five biological replicates were considered. Genes with an adjusted *p*-value ≤ 0.05 (Benjamini and Hochberg, 1995) and a |log_2_FC| ≥ 1.5 were considered differentially expressed (DEs) between pigmented and unpigmented samples.

### 2.5 Sequence Analysis

The analyses of the different sequence characteristics were performed on 14,361 coding genes (34,447 transcripts), 4,937 putative novel lncRNAs, and 218 known lncRNAs. The GC content and the length of transcripts were obtained using the infoseq function of the EMBOSS package version: 6.6.0.0. The exon number was estimated using custom scripts in bash. The abundance was estimated using Cuffnorm. The minimum free energy (ME) was calculated using the RNAfold program included in the ViennaRNA package version 2.4.9 (Zuker and Stiegler, 1981; Lorenz et al., 2011). To make the ME value of the different RNA sequences comparable, we normalized the ME by the sequences’ length, yielding the MEN. Thus, MEN was calculated by dividing the ME value by the transcript’s size and multiplying it by 100. Thus, the MEN value relates the ME estimation to a segment of 100 nucleotides: MEN = (ME/sequence length)*100 (Zhang et al., 2006).

### 2.6 Prediction of Target Genes

Two strategies were used to study the association between lncRNAs and target genes acting in *cis* and *trans*. In the first case, all “neighbor” protein-coding genes showing differential expression (DEGs, *p*-value ≤ 0.01, and |log_2_FC| ≥ 1) were identified in a range of 300 kb upstream and downstream of differentially expressed lncRNAs. Genes that showed significant correlation coefficients at the expression level with neighboring lncRNAs were considered cis target genes (Pearson, r ≥ |0.60|, *p*-value ≤ 0.05).

For potential associations in *trans* with differentially expressed protein-coding genes (DEGs, *p*-value ≤ 0.01 and |log_2_FC| ≥ 1), sequence complementarity with differentially expressed lncRNAs (*p* adjust ≤0.05 and |log_2_FC| ≥ 1.5) was analyzed using LncTar (Li J. et al., 2015). Note that a more stringent threshold was used to detect DE lncRNAs than to detect DE protein-coding genes to obtain a more comprehensive and diverse sample of genes and their functions. The program LncTar identifies potential lncRNA targets by finding the minimum free energy of lncRNA and mRNA pair joint structures. This program was run with a threshold value of ndG −0.08. All predicted interactions of lncRNAs with target mRNAs that showed a significant coexpression correlation (Pearson, r ≥ |0.60|, *p*-value ≤ 0.05) were kept for further analysis.

The functional roles of the target genes were evaluated using the R package EnrichKit (https://github.com/liulihe954/EnrichKit). Different gene set databases, including GO, MeSH, Reactome, InterPro, and MsigDB, were interrogated in the enrichment analysis. Terms significantly enriched within target genes were detected using Fisher’s exact test, a test of proportions based on the hypergeometric distribution.

### 2.7 RNA-Seq Data Validation

The results of the RNA-seq analysis were validated using real-time polymerase chain reaction (qRT-PCR). We selected three differentially expressed lncRNAs and three differentially expressed protein-coding genes for validation. Primer sequences and expected product lengths are listed in Supplementary Table S2. Real-time PCRs were performed using 7.5 µL of SYBR^®^ Green master mix (Maxima SYBR Green qPCR Master Mix (2X), with separate ROX vials, Thermo Scientific™, United States of America), equimolar amounts of forward and reverse primers (200 nM, Operon Biotechnologies GmbH, Cologne, Germany), and 20 ng diluted cDNA (1:7.5 in RNase/DNase free water) in a final volume of 15 µL. Samples were analyzed in duplicate in a 72-disk Rotor-GeneTM 6000 (Corbett Life Sciences, Sydney, Australia). Standard amplification conditions were 10 min at 95°C and 40 cycles of 15 s at 95°C, 30 s at 60°C, and 15 s at 72°C. At the end of each run, dissociation curves were analyzed to ensure that the desired amplicon was detected, discarding contaminating DNA or primer dimers. Gene expression was normalized using *ACTG1* as a housekeeping gene. Normalized gene expression values (ΔCt) were analyzed using a linear model including the pigmentation group as an independent variable. The association between the normalized gene expression and the pigmentation group was tested using a *t*-test. The mean and the range of the log2-fold change for each gene were calculated as log_2_ (2^−ΔΔCt^) using the estimated ΔΔCt value ±standard error.

## 3 Results and Discussion

The aim of this study was to identify and analyze lncRNAs associated with eyelid pigmentation in Hereford cattle. Our findings provide further evidence that lncRNAs are actively involved in pigmentation, as suggested by previous studies not only in cows (Weikard et al., 2013) but also in pigs (Zou et al., 2018), goats (Ren et al., 2016), humans (Zhao W. et al., 2016; Tang et al., 2020) and invertebrate organisms such as *Crassostrea gigas* (Feng et al., 2018).

### 3.1 Identification of Long NonCoding RNAs

**FIGURE 1:**
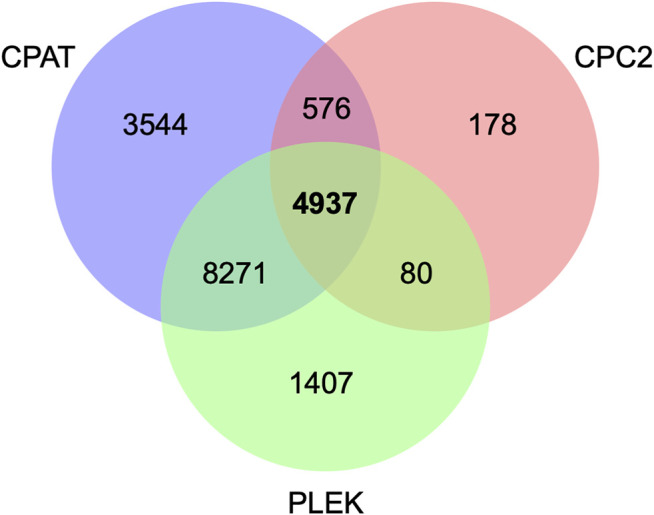
Putative long noncoding RNAs based on CPC2, PLEK, and CPAT tools.

A total of 111,791 transcripts, including 27,806 unannotated transcripts, were obtained after mapping the sequencing reads to the latest bovine genome reference. Among the novel transcripts, 218 were detected in the ALDB and NONCODE databases and were therefore considered known lncRNAs (Supplementary Data Sheet S1). After training, a coding potential cutoff of 0.36 was selected for the CPAT. This value maximizes specificity and sensitivity (98.3%) (see Supplementary Figure S1), similar to other studies (Billerey et al., 2014; Gupta et al., 2019). All transcripts with a score below 0.36 were retained as potential long noncoding RNAs. After integrating the results from CPC2, CPAT, and PLEK, we identified a total of 4,937 unannotated transcripts as putative lncRNAs (Figure 1, Supplementary Figure S2).

Based on RNA-seq analysis of 18 different tissues, Koufariotis et al. (2015) identified 9,778 lncRNAs in cattle. Of special interest, seven lncRNAs were found to be differentially expressed between white and black skin samples (Koufariotis et al., 2015). A recent paper reported a total of 1,535 expressed lncRNAs encoded by 1,183 putative noncoding genes in bovine oocytes (Wang et al., 2020). More specifically, Weikard et al. (2013) identified 4,848 lncRNAs that are associated with pigmentation in cattle, supporting the idea that lncRNAs play an important role in skin pigmentation. Notably, this number of lncRNAs is similar to the 4,937 transcripts that we reported in the present study, although only 314 were in common. The difference between studies could be explained in terms of the analytical pipeline used to identify putative lncRNAs in each case. Note that at the time of writing this manuscript, there is no widely accepted pipeline to identify lncRNAs.

### 3.2 Comparison Between mRNAs, Novel Long NonCoding RNAs, and Known Long NonCoding RNAs

We identified a total of 4,937 novels and 218 known lncRNAs in our RNA-seq dataset. The lncRNAs were characterized as novel isoforms (51.5%), sense exon overlap (14.5%), antisense intron overlap (12.3%), intergenic (16.5%), and antisense exon overlap (5.2%) forms (Supplementary Figure S3, Supplementary Data Sheet S1). Of the 51.5% characterized as novel isoforms, 94% were generated from regions that harbor protein-coding genes while 6% were novel isoforms from known lncRNAs. Note that similar results were recently reported by Alexandre et al. (2020) working on lncRNAs associated with feed efficiency in cattle.

**FIGURE 2:**
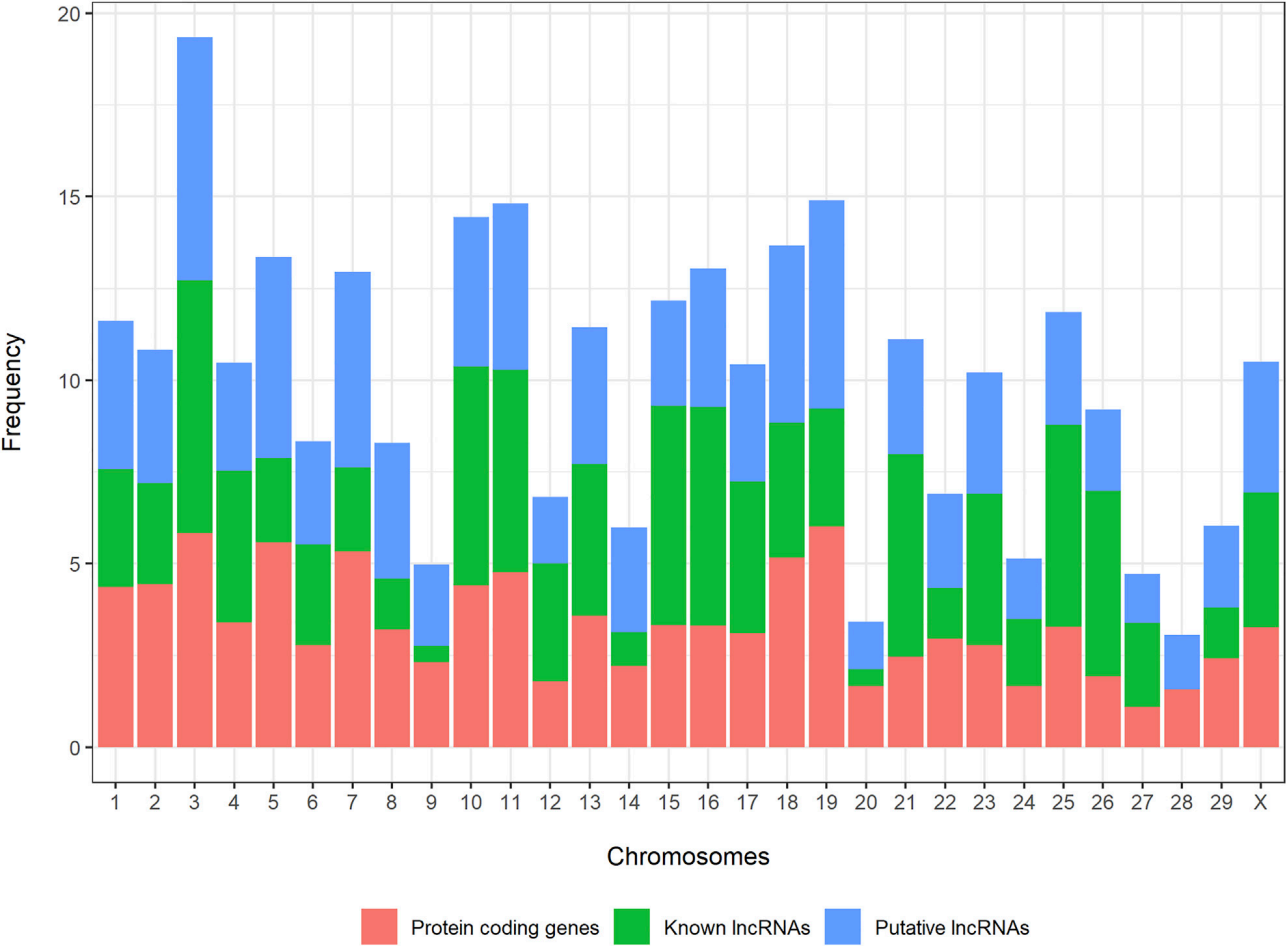
Distribution of novel long noncoding RNAs, known long noncoding RNAs, and protein-coding genes across the bovine genome.

Novel and known lncRNAs were evenly distributed across the whole genome, ranging from 1.3% (novel lncRNAs) and 0.45% (known lncRNAs) in BTA20 to 6.6% (novel lncRNAs) and 6.8% (known lncRNAs) in BTA3 (Figure 2).

**FIGURE 3:**
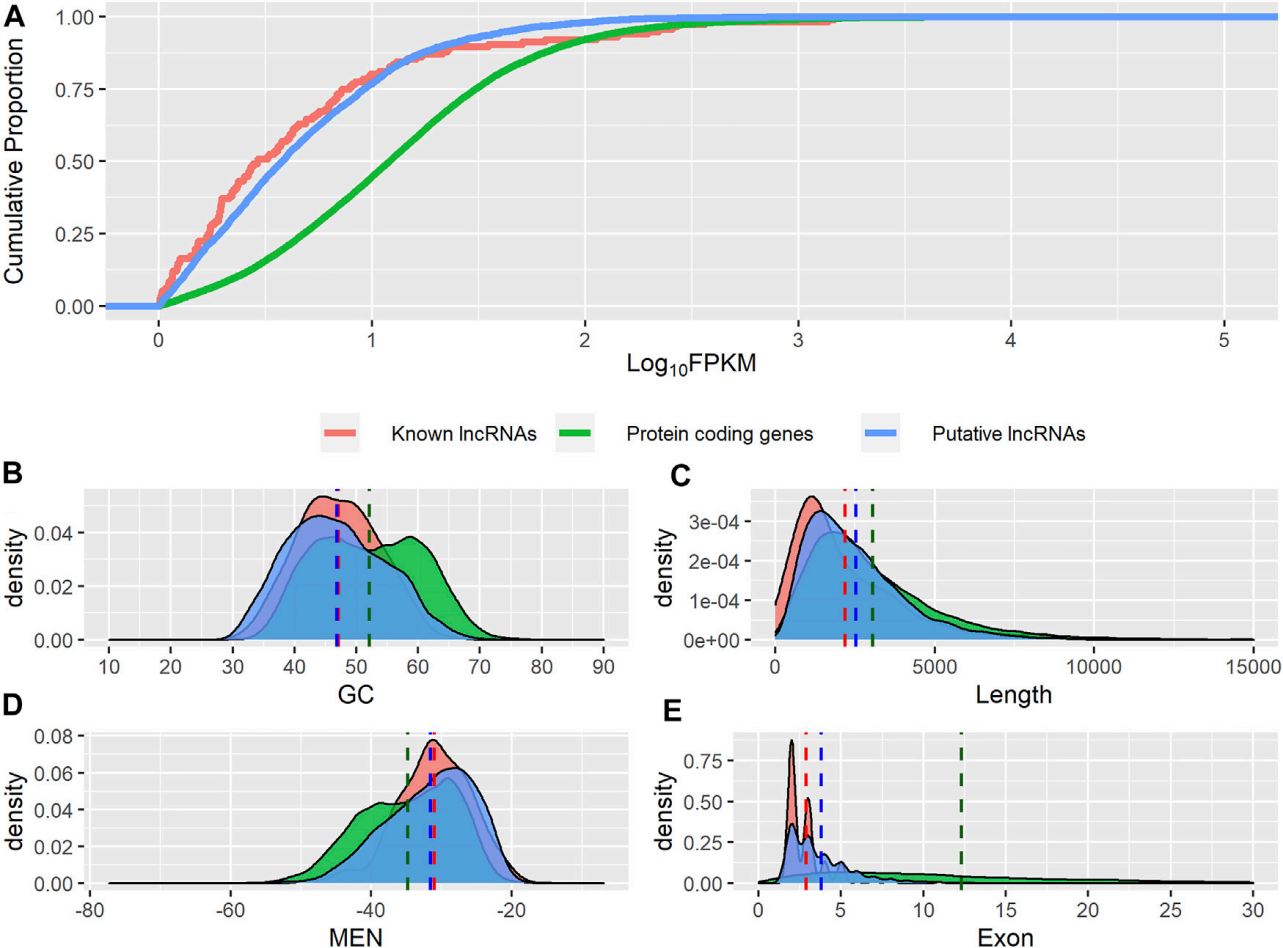
Genomic features of putative lncRNAs, known lncRNAs, and protein-coding genes. **(A)** Comparison of expression level. **(B)** Distribution of guanine-cytosine content (GC). **(C)** Distribution of transcripts length. **(D)** Distribution of normalized minimum free energy (MEN). **(E)** Distribution of number of exons.

We analyzed the guanine-cytosine content (GC), normalized minimum free energy (MEN), transcript length, exon number, and expression level of all putative novel lncRNAs and compared these metrics with those from known lncRNAs and protein-coding genes. Both groups of lncRNAs displayed significantly lower GC content (Figure 3B), shorter length (Figure 3C), and higher MEN (Figure 3D) than protein-coding genes (*p*-value ≤ 0.05, Wilcoxon test, Table 1). We found no significant differences in the GC content nor in the MEN between the novel and known lncRNAs (*p*-value = 0.37 and *p*-value = 0.55, Wilcoxon test, respectively, Table 1). These results agree with previous studies that showed that lncRNAs have some unique sequence features compared to coding genes (Niazi and Valadkhan, 2012).

**TABLE 1:** Comparison of novel lncRNAs, known lncRNAs, and protein-coding genes.

Category	GC	Length	MEN	Exons	Expression
**Novel lncRNAs**	46.9 ± 0.11^A^	2532 ± 24^A^	−31.5 ± 0.09^A^	3.8 ± 0.03^A^	0.34 ± 0.01^A^
**Known lncRNAs**	47 ± 0.44^A^	2199 ± 15^B^	−31.0 ± 0.35^A^	2.9 ± 0.09^B^	0.21 ± 0.05^B^
**Protein-coding genes**	52 ± 0.05^B^	3056 ± 11^C^	−34.7 ± 0.04^B^	12.3 ± 0.06^C^	0.98 ± 0.01^C^

Expression: log_10_ FPKM.

Different letters represent significant differences (*Wilcox test*, *p*-value ≤ 0.01).

Both novel and putative lncRNAs showed significantly higher MEN values than protein-coding genes (*p*-value ≤ 0.05, Wilcoxon test, Table 1). The sequence itself seems to contribute considerably to MEN since sequences with the same GC content can potentially present different folding energy values based on their secondary structure (Niazi and Valadkhan, 2012). It can be inferred that lncRNAs have a more flexible structure than mRNAs, which may reflect a higher potential to interact with other molecules. Indeed, it has been shown that lncRNAs have GC and MEN values similar to those of 3′ UTR sequences, suggesting that lncRNAs have regulatory functions (Niazi and Valadkhan, 2012).

We found that the novel lncRNAs were significantly shorter than the known lncRNAs (*p*-value ≤ 0.05, Wilcoxon test, Table 1). Both groups of noncoding transcripts showed lower expression levels (Figure 3A) and a lower number of exons (Figure 3E) than protein-coding genes (*p*-value ≤ 0.05, Wilcoxon test).

Overall, our results showed that lncRNAs are characterized by a lower number of exons, lower GC content, lower expression level, and higher normalized minimum free energy and tend to be shorter than protein-coding sequences (Derrien et al., 2012; Harrow et al., 2012; Billerey et al., 2014). Note that these sequence feature analyses support the reliability of the putative novel lncRNAs identified. We believe that the marginal differences observed in the length, expression level, and lower number of exons between the novel and known lncRNAs could be explained by the limited catalog of lncRNAs in cattle (Kosinska-Selbi et al., 2020). However, we could not completely discard the possibility that some of our novel lncRNAs are either pseudogenes or misidentified coding sequences.

### 3.3 Expression Analysis

We found 65 differentially expressed protein-coding genes (*p* adjusted ≤0.05 and |log_2_ FC| ≥1.5) between the pigmented and unpigmented samples (Appendix S2). Among them, *MC1R*, *TYR*, *PMEL*, *DCT*, *MLANA,* and *KIT* showed upregulated expression in pigmented eyelid samples (Jara et al., 2020). These genes are key for the generation, storage, and distribution of melanin (D’Mello et al., 2016). Gene *KIT* encodes a receptor of tyrosine kinase and is considered a proto-oncogene, involved in the development of melanocytes, including migration, survival, proliferation, and differentiation (Grichnik, 2006). Previous studies showed the important role of *KIT* in UVB-induced epidermal melanogenesis in humans (Yamada et al., 2013) and in the survival of melanocytes (Mort et al., 2015). Here, *KIT* showed higher expression in pigmented eyelid samples, suggesting an important role in pigmentation. Interestingly, our analyses suggest that lncRNAs do not interact directly with *KIT*, and hence, it seems this gene is not directly regulated by these non-coding elements. Moreover, we identified a total of 27 differentially expressed lncRNAs (*p* adjusted ≤0.05 and |log_2_FC| ≥ |1.5|); twenty-four were putative novel lncRNAs, and three were classified as known lncRNAs. Eight lncRNAs showed upregulated and 19 lncRNAs showed downregulated expression in the pigmented eyelid samples. The top lncRNA with significantly downregulated expression was TCONS_00073858 (novel) with log_2_FoldChange = −10.3, while the top lncRNA with upregulated expression was TCONS_00088900 (novel) with log_2_FoldChange = 7.7.

### 3.4 Association of Long NonCoding RNAs With Putative Target Genes Acting in cis

We analyzed potential target mRNAs of lncRNAs acting in *cis*, that is, protein-coding genes within 300 kb upstream and downstream of the location of significant differentially expressed lncRNAs. We found four putative target genes, namely, *CXCL13*, *FABP4*, *GPR143*, and *UGT1A1,* in the flanking regions of four differentially expressed lncRNAs, TCONS_00094682 (novel), TCONS_00021379 (novel), TCONS_00109545 (novel), and TCONS_00078693 (novel), respectively (Table 2). The lncRNAs TCONS_00094682 and TCONS_00021379 showed downregulated expression in the pigmented samples and acted at the *cis* level with the *CXCL13* and *FABP4* genes, respectively. The *CXCL13* gene encodes a homeostatic chemokine that traffics B cells and is involved in the immune response (Müller et al., 2002). Fatty acid transporters, such as *FABP4,* were recently shown to increase their expression during metastatic melanoma development, suggesting a higher uptake of fatty acids and metabolic reprogramming that serves as a signature for this condition (Lee et al., 2020). In contrast, the lncRNAs TCONS_00109545 and TCONS_00078693 showed upregulated expression in the pigmented samples. The lncRNA TCONS_00109545 interacts at the *cis* level with the *GPR143* gene, which plays a critical role in retinal health and development (McKay, 2019). Mutations in *GPR143* have been associated with the ocular albinism type 1 (OA1) phenotype (Gao et al., 2020), a genetic disorder characterized by reduced ocular pigmentation. Finally, lncRNA TCONS_00078693 was correlated with the expression of *UGT1A1,* a gene implicated in retinoic acid binding. Retinoid signaling is affected in early carcinogenesis (Tang and Gudas, 2011), and retinoic acid is often used to prevent photoaging of human skin, preventing melanocytic and keratinocytic atypia (Cho et al., 2005). Note that all these lncRNAs showed a positive correlation with their target genes, and hence, we hypothesize that these lncRNAs interact at the *cis* level, promoting the expression of these genes through the recruitment of proteins that enable transcription loops (Long et al., 2017; Li et al., 2019; Gil and Ulitsky, 2020).

**TABLE 2:** LncRNAs and target genes.

LncRNAs	Target genes	Correlation (r)
TCONS_00094682	*CXCL13*	0.99
TCONS_00021379	*FABP4*	0.97
TCONS_00109545	*GPR143*	0.96
TCONS_00078693	*UGT1A1*	0.98

### 3.5 Association of Long NonCoding RNAs With Putative Target Genes Acting in Trans

We analyzed the potential target mRNAs of lncRNAs acting in *trans* from a total of 273 differentially expressed genes that showed sequence complementarity with at least one of the twenty-seven differentially expressed lncRNAs. Of these 273 genes, 193 showed sizable expression correlations with significant lncRNAs (Pearson, r > |0.60|, *p*-value < 0.05) (Supplementary Data Sheet S3); hence, they were classified as potential *trans* target genes. We identified target genes involved in melanogenesis, melanosome development (Lin and Fisher, 2007; Kubic et al., 2008; Schmutz and Dreger, 2013), pigmentation processes (Lalueza-Fox et al., 2007; Enriqué Steinberg et al., 2013), tumor pathways (Hoashi et al., 2005), innate immunity and inflammatory signaling (Ito et al., 2015), such as *MC1R, PMEL, MLANA*, *PAX3, IGFBP2, FGF23,* and *TREM-2*. Interestingly, many of these potential *trans* target genes were reported previously as differentially expressed in pigmented versus non-pigmented samples (Jara et al., 2020) (Supplementary Figure S4, Supplementary Data Sheet S3). The genes *MC1R, PMEL, MLANA*, and *PAX3* showed upregulated expression in the pigmented samples and interacted with lncRNAs with up- and down-regulated expression (Supplementary Data Sheet S2, S3). *MC1R* is a key pigmentation gene, and its activation in melanocytes stimulates melanogenesis, particularly eumelanogenesis (Beaumont et al., 2007; Cheli et al., 2010; Enriqué Steinberg et al., 2013). The *PMEL* gene is a key component of mammalian melanosome biogenesis (Clark et al., 2006), and it is required for the generation of cylindrical melanosomes in zebrafish (Watt et al., 2013; Burgoyne et al., 2015). Mutations in *PMEL* have been shown to regulate hypopigmented phenotypes in vertebrates (Kwon et al., 1995; Kerje et al., 2004). The transcription factor *PAX3* interacts with lncRNA TCONS_00088900 and regulates the expression of *MITF* (Lin and Fisher, 2007), a gene associated with ambilateral circumocular pigmentation in cattle (Pausch et al., 2012). *MC1R* and *MLANA* expression levels showed significant positive correlations with the novel lncRNAs TCONS_00088900 and TCONS_00091890, while *PMEL* also showed a significant positive correlation with TCONS_00091890. *MLANA* encodes a protein (MART-1) that is localized in melanosomes (De Maziere et al., 2002). Interestingly, *MLANA* interacts with *PMEL* and regulates its expression, stability, trafficking, and processing (Hoashi et al., 2005). *MLANA* showed negative correlations with the lncRNAs ALDBBTAT0000004273 and TCONS_00106295. LncRNAs have different ways of affecting the transcription of target genes *in trans*, for example, by stabilizing the mRNA (Cao et al., 2017; Long et al., 2017; Li et al., 2019). The participation of lncRNAs in mRNA stabilization and in increasing the expression of specific genes has been recently discussed (Li et al., 2019). From our results, we can infer that ALDBBTAT0000004273 and TCONS_00106295 could be involved in reducing the stability of the mRNA encoded by the *MLANA* gene and consequently contributing to reduced expression in unpigmented samples. Additionally, the lncRNAs could be involved in the stabilization of the mRNAs of certain genes responsible for pigmentation, such as *PEML* and *MC1R*, and thus enhance their expression levels in pigmented eyelids. The genes *IGFBP2, FGF23,* and *TREM-2* showed downregulated expression in the pigmented samples and interacted with lncRNAs with up- and down-regulated expression (Supplementary Data Sheet S2, S3).The *FGF23* gene may act as a proinflammatory cytokine (Ito et al., 2015), suggesting a connection between *FGF23* and inflammatory processes (Courbebaisse and Lanske, 2018). In fact, high expression of *FGF23* is associated with an increased risk of mortality, likely because of its contribution to decreased host defense response to infection, inflammation, and anemia (Courbebaisse and Lanske, 2018). *FGF23* showed positive correlations with the ALDBBTAT0000004273 and TCONS_00106295 lncRNAs. The *IGFBP2* gene is involved in tumor pathways (Pickard and McCance, 2015) and showed positive correlations with the lncRNAs ALDBBTAT0000001157 and TCONS_00106295. The fatty acid-binding protein 7 (*FABP7*) gene was also described as a key regulator of cancer metastasis (Cordero et al., 2019), and its expression was upregulated in pigmented samples (Jara et al., 2020). This gene showed significant positive and negative correlations with several lncRNAs. The *TREM-2* gene showed a positive correlation with lncRNA TCONS_00073858, which is implicated in innate immunity and inflammatory signaling (Sharif and Knapp, 2008). TREM (*TREM-1/TREM-2*) gene expression is lower in cutaneous melanoma versus control samples (Nguyen et al., 2015), and these genes could have prognostic and therapeutic value in the treatment of melanoma (Nguyen et al., 2015; Nguyen et al., 2016). From our results, it can be speculated that the lncRNAs described here are involved in stabilizing the mRNAs of genes involved in the immune system and cancer, such as *FGF23* (Courbebaisse and Lanske, 2018), *IGFBP2* (Pickard and McCance, 2015) and *TREM-2*.

### 3.6 Gene Set Enrichment Analysis of Putative Target Genes

**FIGURE 4:**
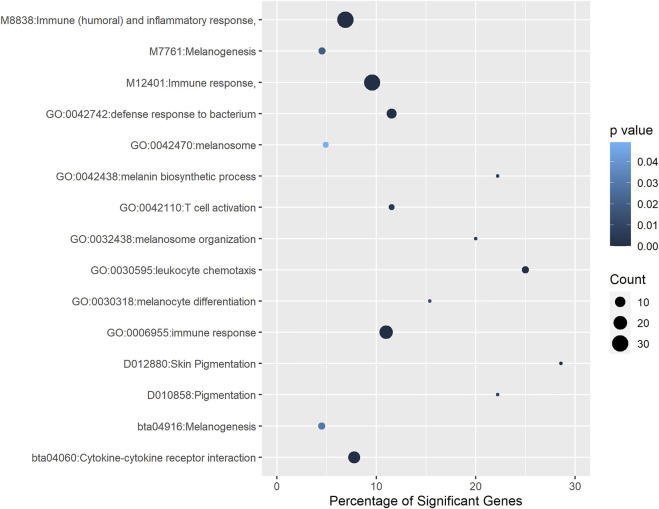
Functional terms and pathways significantly enriched with genes associated with eyelid pigmentation. Different annotation databases, including GO, Medical Subject Headings, InterPro, Reactome and MSigDB, were used.

A total of 193 genes were identified as putative targets of DE lncRNAs in pigmented samples. To identify enriched functions among them, we performed a gene set enrichment analysis using the annotation retrieved from different databases, including GO, KEGG, MeSH, InterPro, Reactome, and MSigDB. Figure 4 shows the most relevant biological terms and pathways associated with eyelid pigmentation. The most significant functional terms were related to skin pigmentation, such as *melanosome* (GO:0042470), *melanin biosynthetic process* (GO:0042438), *melanosome organization* (GO:0032438), *melanocyte differentiation* (GO:0030318), *melanogenesis* (bta04916), *skin pigmentation* (D012880), *pigmentation* (D010858) and *melanogenesis* (M7761) (Figure 4, Supplementary Data Sheet S4). Notably, we found several significant terms related to the inflammatory response and infectious and tumoral pathways: *immune response* (GO:0006955), *defense response to bacterium* (GO:0042742), *leukocyte chemotaxis* (GO:0030595), *cytokine-cytokine receptor interaction* (bta04060), *T cell activation* (GO:0042110), *cytokine-cytokine receptor interaction* (bta04060), *immune response* (M12401), and *immune (humoral) and inflammatory response* (M8838) (Figure 4, Supplementary Data Sheet S4). Our findings indicate that target genes of lncRNAs with up- and down-regulated expression in eyelid skin are associated not only with pigmentation or melanogenesis but also with the immune response.

### 3.7 Validation of Gene Expression Using Quantitative Real-Time Polymerase Chain Reaction

**FIGURE 5:**
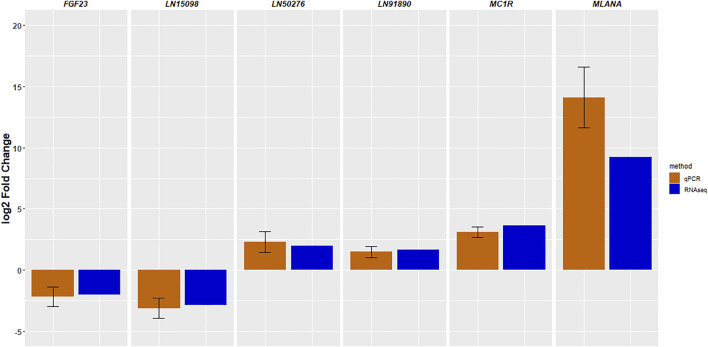
Validation of RNA-sequencing results by quantitative RT-PCR. The data are shown as the mean ± standard error.

We validated the findings of the RNA-seq experiment using qRT-PCR. Three lncRNAs and three protein-coding genes were evaluated. Figure 5 shows the log2-fold differences in gene expression measured by both RNA-Seq and qRT-PCR, confirming that the six genes showed very similar patterns of abundance with both methods.

## 4 Conclusion

In this work, we described the expression patterns of lncRNAs in the eyelid skin of cattle. We predicted 4,937 putative novel lncRNAs, mapped them to the latest bovine reference genome, and compared their sequence features to those of known lncRNAs and protein-coding genes. A total of 27 lncRNAs were identified as differentially expressed between the pigmented and unpigmented samples. Potential associations were found between specific lncRNAs and putative target genes directly implicated in pigmentation, immune responses, and cancer development. Overall, our study enriches the catalog of lncRNAs in *B. taurus*, specifically those related to the regulation of eyelid skin pigmentation. Future functional studies should further evaluate the biological functions of these significant lncRNAs.

## Data Availability

The original contributions presented in the study are included in the article/Supplementary Material, further inquiries can be directed to the corresponding author.

## Abbreviations

CPC2, Coding Potential Calculator 2; CPAT, Coding-Potential Assessment Tool; PLEK, Predictor of long noncoding RNAs and messenger RNAs based on an improved k-mer scheme; FPKM, fragments per kilobase of transcript per million mapped reads; GO, Gene Ontology; KEGG, Kyoto Encyclopedia of Genes and Genomes; MeSH, Medical Subject Headings; MSigDB, Molecular Signatures Database; lncRNAs, long noncoding RNAs; qRT-PCR, real-time quantitative polymerase chain reaction; NCBI, National Center for Biotechnology Information.
