# Supplementary material for: Identification of Long Noncoding RNAs Involved in Eyelid Pigmentation of Hereford Cattle
Source: Front Genet. 2022 May 4;13:864567. doi: 10.3389/fgene.2022.864567 (PMC9114348; doi:10.3389/fgene.2022.864567)
Supplement: Supplementary file 1 [file Table1.DOCX]

**Supplementary Table S1. RNA sequencing statistics for 11 samples***

|  | Raw reads | Clean reads  Pairs | Clean reads single | Clean reads | Read  length | Mapped  reads | Concordantly  mapped pairs | Concordant pair  align rate (%) | Overall alignment  rate (%) |
| --- | --- | --- | --- | --- | --- | --- | --- | --- | --- |
| A1 | 66,190,422 | 49,659,736 | 5,195,825 | 54,855,561 | 150 | 51,068,534 | 22,566,547 | 90.88 | 93.10 |
| B2 | 56,997,366 | 42,604,208 | 4,494,974 | 47,099,182 | 150 | 43,981,216 | 19,168,165 | 89.98 | 93.58 |
| C3 | 78,365,632 | 59,645,918 | 5,926,765 | 65,572,683 | 150 | 60,274,410 | 25,902,322 | 86.85 | 91.92 |
| D4 | 70,970,338 | 54,647,578 | 5,067,503 | 59,715,081 | 150 | 55,588,769 | 24,521,961 | 89.75 | 93.09 |
| E5 | 55,098,260 | 42,104,804 | 4,100,191 | 46,204,995 | 150 | 43,363,388 | 19,214,643 | 91.27 | 93.85 |
| F6 | 48,859,594 | 36,472,860 | 3,937,134 | 40,409,994 | 150 | 37,334,793 | 16,221,722 | 88.95 | 92.39 |
| H8 | 57,126,090 | 43,894,802 | 4,126,327 | 48,021,129 | 150 | 44,501,180 | 19,568,457 | 89.16 | 92.67 |
| I9 | 53,490,934 | 40,520,864 | 4,119,137 | 44,640,001 | 150 | 41,207,185 | 18,144,971 | 89.56 | 92.31 |
| J10 | 59,453,408 | 45,237,500 | 4,441,703 | 49,679,203 | 150 | 45,898,616 | 20,258,030 | 89.56 | 92.39 |
| K11 | 56,873,562 | 43,781,722 | 4,115,550 | 47,897,272 | 150 | 46,584,189 | 19,782,792 | 90.37 | 93.77 |
| W12 | 46,489,988 | 35,008,348 | 3,648,025 | 38,656,373 | 150 | 38,656,373 | 15,744,954 | 89.95 | 92.80 |

*A1, E5, J10, K11, and W12 are pigmented samples while B2, C3, D4, F6, H8, and I9 are unpigmented eyelid samples.
