# Supplementary figures and images for: Identification of Long Noncoding RNAs Involved in Eyelid Pigmentation of Hereford Cattle
Source: Front Genet. 2022 May 4;13:864567. doi: 10.3389/fgene.2022.864567 (PMC9114348; doi:10.3389/fgene.2022.864567)

Supplementary Figure S2. Bioinformatic pipeline for the identification of putative lncRNAs.

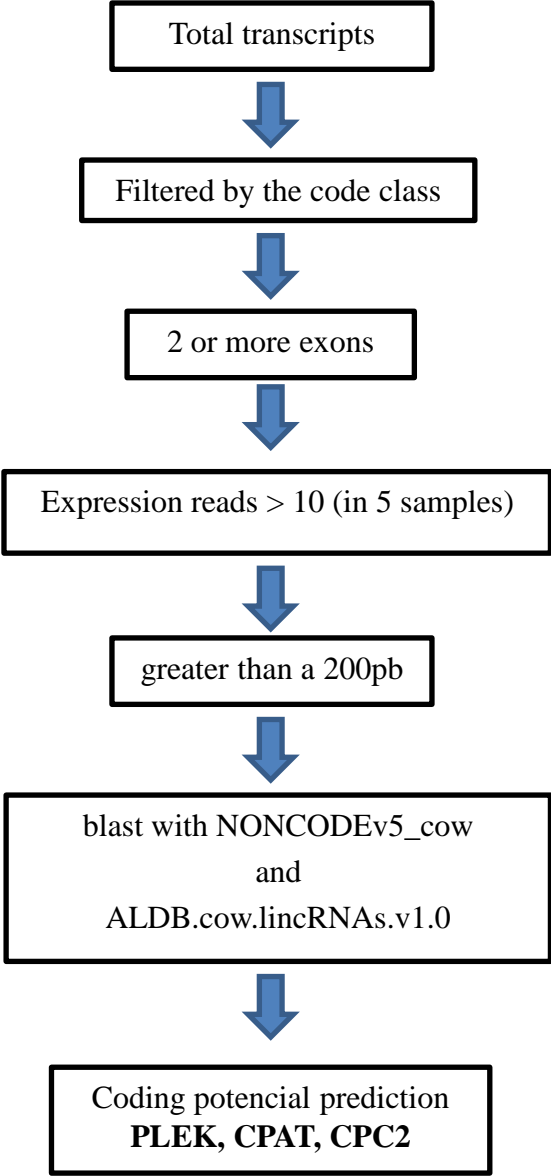

Supplement: Supplementary file 4 [file Image2.PDF]

Supplementary Figure S3. Distribution of classes among putative and known lncRNAs.

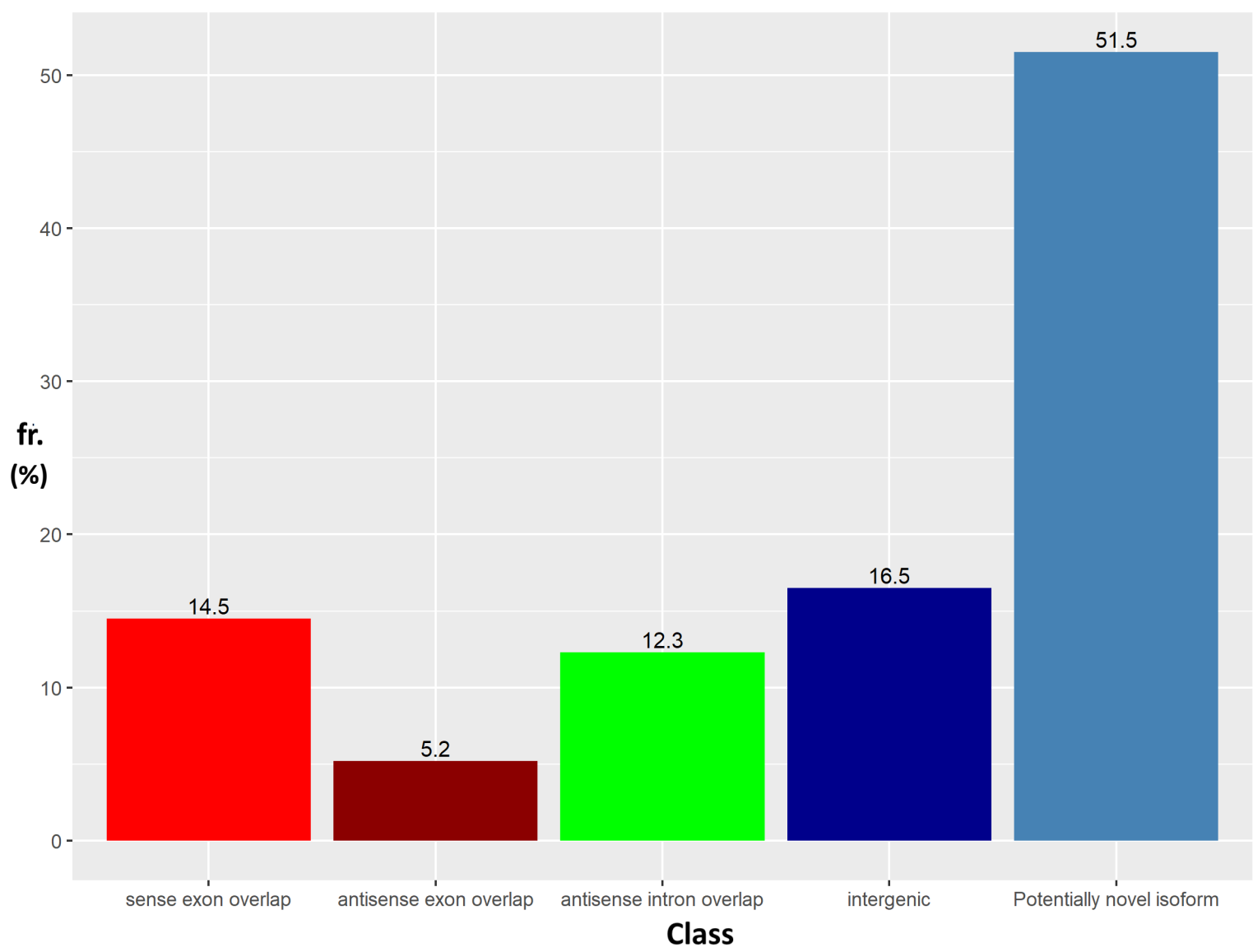

Supplement: Supplementary file 5 [file Image3.PDF]

Supplementary Figure S1. Performance evaluation using 10-fold cross-validation.

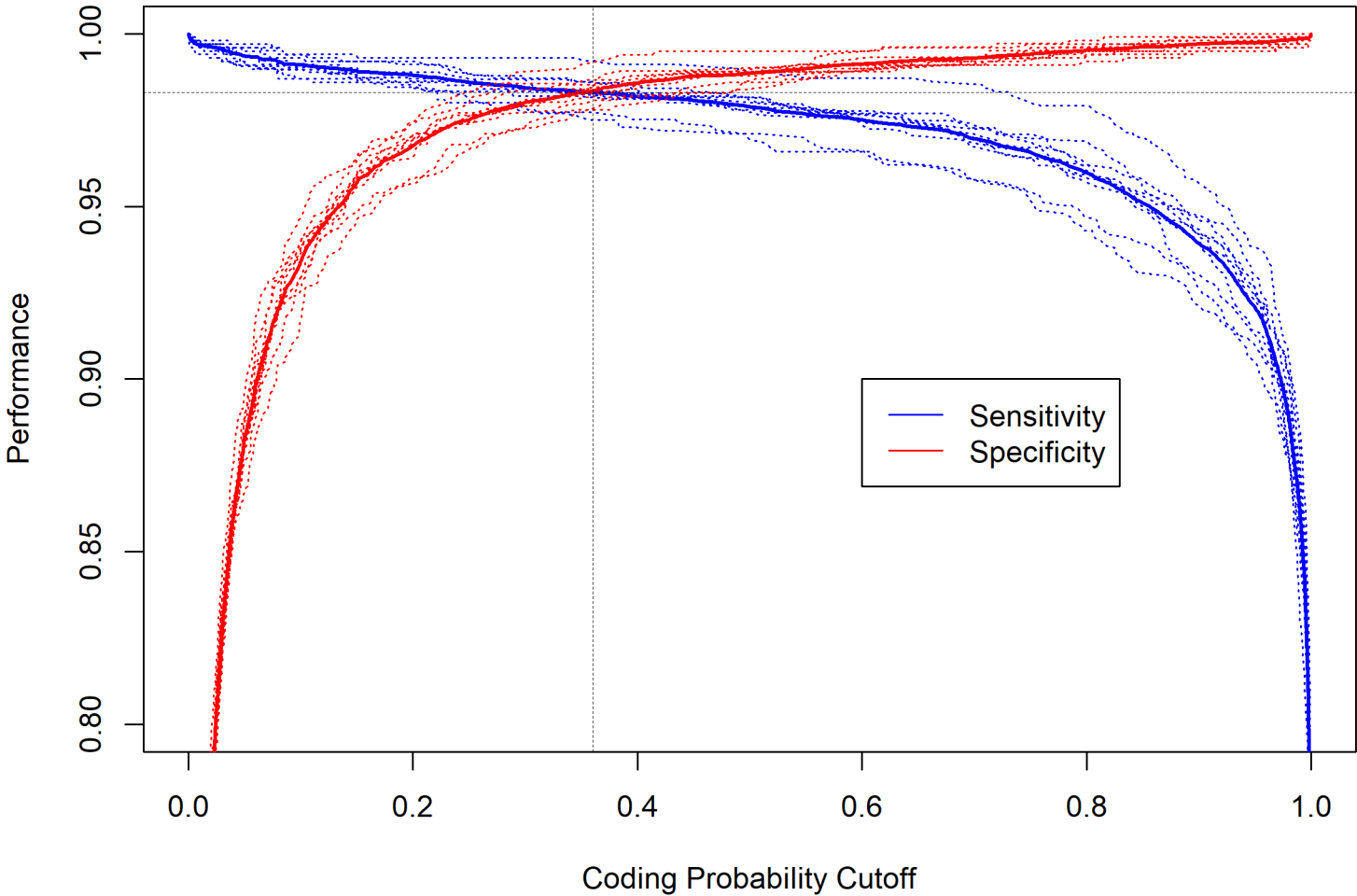

Supplement: Supplementary file 8 [file Image1.PDF]
