# Supplementary material for: Identification of Long Noncoding RNAs Involved in Eyelid Pigmentation of Hereford Cattle
Source: Front Genet. 2022 May 4;13:864567. doi: 10.3389/fgene.2022.864567 (PMC9114348; doi:10.3389/fgene.2022.864567)
Supplement: Supplementary file 7 [file Table2.DOCX]

**Supplementary Table S2. Primer sequences and expected amplicon sizes used for real-time reverse transcription–polymerase chain reaction (RT-PCR) assays.**

| Gene ^a^ | Accession no | Primer sequence^b^ | Length (bp) |
| --- | --- | --- | --- |
| TCONS_00050276 |  | F-GGATTCCGGACTCAAAAGCC | 99 |
|  |  | R-GGGGTAAGTGACAGGCAGAT |  |
| TCONS_00091890 |  | F-ATTCTGGTTGGGGCATGAGA | 124 |
|  |  | R-TTGCTCACCATCCCATCAGT |  |
| ALDBBTAT0000001157 |  | F-AGCGATGAGAGACCAAGGAG | 128 |
|  |  | R-GTGATTCTCGGCAACCTTGG |  |
| ACTG1 | ENSBTAG00000006189 | F-GCCAACCGTGAGAAGATGAC | 90 |
|  |  | R-GCAGGAGTGTTGAACGTCTC |  |
| MC1R | ENSBTAG00000023731 | F-GTCCTCTACGTCCACATGCT | 96 |
|  |  | R-GCCAAAGCCCTGATGAATGG |  |
| MLANA | ENSBTAG00000007440 | F- CCACTCTTATGTCACGGCTG | 112 |
|  |  | R- TTCGGTATCCACTTCGTCGT |  |
| FGF23 | ENSBTAG00000030343 | F- ATGATCAGGTCGGAGGATGC | 117 |
|  |  | R- GCTCTCCGGACTGAAGTGAT |  |

ACTG1 Actin gamma 1

b F= forward, R= reverse

ACTG1=Actin gamma 1.

** Forward and reverse, respectively.
