# Supplementary material for: Identification of Long Noncoding RNAs Involved in Eyelid Pigmentation of Hereford Cattle
Source: Front Genet. 2022 May 4;13:864567. doi: 10.3389/fgene.2022.864567 (PMC9114348; doi:10.3389/fgene.2022.864567)

Supplementary Figure S4. Coexpression heatmap between lncRNAs and gene targets. Positive and negative correlations between lncRNAs and target genes are shown in blue and red, respectively.

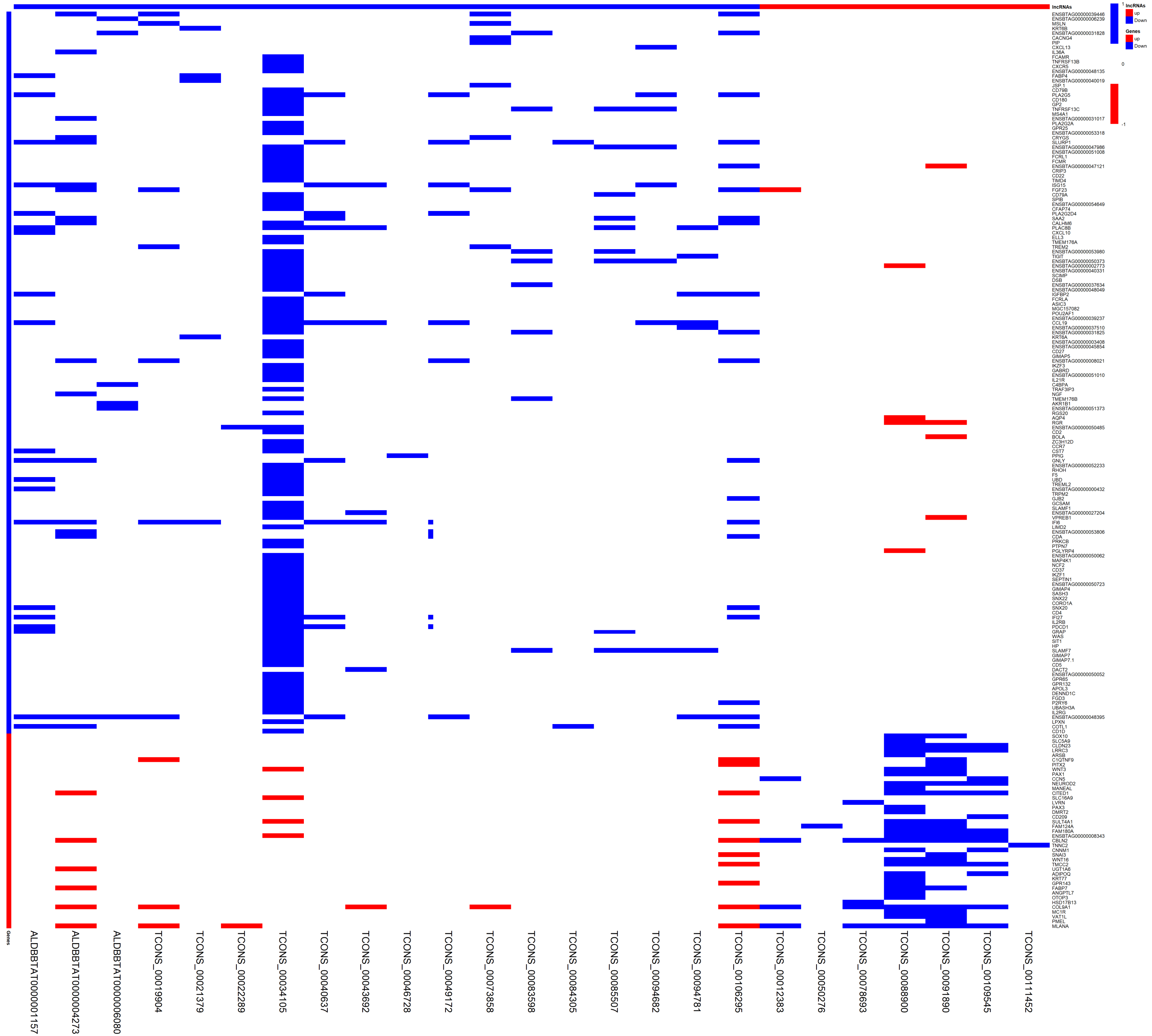

Supplement: Supplementary file 3 [file Image4.PDF]
